# Modulation of Plasma Proteomic Profile by Regular Training in Male and Female Basketball Players: A Preliminary Study

**DOI:** 10.3389/fphys.2022.813447

**Published:** 2022-03-14

**Authors:** Rosamaria Militello, Gabriella Pinto, Anna Illiano, Simone Luti, Francesca Magherini, Angela Amoresano, Pietro Amedeo Modesti, Alessandra Modesti

**Affiliations:** ^1^Department of Biomedical, Experimental and Clinical Sciences “Mario Serio,” University of Florence, Florence, Italy; ^2^Istituto Nazionale Biostrutture e Biosistemi, Rome, Italy; ^3^Department of Chemical Sciences, Polytechnic and Basic Sciences School, University of Naples Federico II, Naples, Italy; ^4^Department of Experimental and Clinical Medicine, University of Florence, Florence, Italy

**Keywords:** plasma proteome, exerkines, regular training, basketball, sex differences

## Abstract

Monitoring fatigue and recovery during training periods contributes to identifying the best training methods to achieve sports performance. To date, little is known about sex-related differences in sports adaptations. The aim of the present study is to identify sex-related sports adaptation proteins in female basketball players and male basketball players using proteomics approach on plasma samples withdrawn from athletes during in-season training period but far from a competition. A cohort of 20 professional basketball players, 10 female (BF) and 10 male (BM), and 20 sedentary male (10 CM) and female (10 CF) as control, of comparable age and BMI, were involved in this study. Protein profiles of plasma samples obtained from BM, BF, CM, and CF were analyzed by two-dimensional electrophoresis (2-DE). Differentially expressed proteins were identified by mass spectrometry. The computational 2-DE gel image analysis pointed out 33 differentially expressed protein spots (ANOVA *p*-value < 0.05) and differences between male and female basketball players are more evident among the players than controls. The expression profile of 54.5% of the total proteins is affected by sports activity. Furthermore, 14 proteins are differentially expressed in basket female players in comparison with their relative controls while seven are differentially expressed in basket male players in comparison with their controls. In conclusion, we identify in female athletes a reduction in proteins related to transcription regulation, most of these modulate chronic inflammation confirming the anti-inflammatory effect of regular training in female muscle metabolism. In male and female athletes, we found a decrease in Transthyretin involved in muscle homeostasis and regeneration and Dermcidin a stress-induced myokine linked to inflammatory and it will be interesting to fully understand the role of its different isoforms in male and female skeletal muscle contraction.

## Introduction

Physical activity plays a key role in well-being and keeps the body fit (Alzharani et al., 2020). Inducing metabolic changes in the whole organism, physical activity, and more precisely training in sports practice, leads to the activation of adaptive responses to establish a new equilibrium with beneficial effects for the entire body and performance (Magherini et al., 2019). It is well known, for example, that lactate mediates exercise-induced adaptations (Nalbandian and Takeda, 2016) but also oxidative stress, hormone signaling linked to inflammation appear to be crucial in performance (Luti et al., 2020).

Differently from myokines and adipokines (peptide and miRNAs), which are expressed, produced, and secreted by skeletal muscle and fat depots, respectively (Kirk et al., 2020), the term “exerkines” refers to the total of all humoral exercise factors (peptides and RNA species) that are expressed, produced and secreted by all tissues and organs into the circulation to promote crosstalk between organs and potentiate the systemic benefits of exercise (Nederveen et al., 2020). Sometimes when training is excessive, prolonged, and at high intensity and when it is not followed by an adequate rest and recovery period, it can lead a negative effect on health and in particular on the redox state and inflammation by compromising the immune response (Pedersen, 2006; La Gerche and Heidbuchel, 2014). This can also occur in professional athletes who increase the training loads to improve their performance. When the workload becomes excessive it is easy to reach an opposite effect characterized by fatigue, lack of energy, and a sense of exhaustion (Wan et al., 2017); in the acute phase, this phenomenon is defined with the term overreaching and is reversible because it can be resolved with an adequate rest period. On the contrary, when it persists over time it can lead to chronic fatigue syndrome (CFS) and overtraining syndrome, a phenomenon characterized by fatigue, an increase in pro-inflammatory markers, and a decrease in performance, with harmful consequences for health (Lehmann et al., 1997).

For this reason, is important to identify markers, such as oxidative stress or signal molecules of inflammation, to monitor the level of training to avoid phenomena, such as overtraining, or to increase training loads to improve performance.

In our opinion, basketball is a good model of complete sports since it is characterized by frequent movement changes due to the transition between offense and defense (McInnes et al., 1995). These transitions differ in terms of movement pattern (e.g., running, jumping, and mixing), intensity, distance, frequency, duration while jumps unlike other team sports occur approximately every minute (Ben Abdelkrim et al., 2007; Scanlan et al., 2011). Because of these frequent changes, there are periods of high-intensity activity interspersed with periods of low or moderate intensity, characterized by aerobic and anaerobic intermittent demands (Ben Abdelkrim et al., 2007). However, the anaerobic pathway becomes preponderant during high-intensity actions (Narazaki et al., 2009). In conclusion in our opinion, this sports is useful to study modifications in biological mechanisms in athletes during the in-season training period. In a previous study submitted to Metabolites (under revision), we have highlighted that male basketball athletes are less subject to oxidative stress in comparison to soccer, suggesting that this could be due to the right training methods in basketball as well as the lower duration and intensity of physical effort in the basket. Moreover, we recently have reported that basketball female athletes have a greater biological antioxidant capacity and a lower plasma oxidative species in comparison to controls (Militello et al., 2021).

In recent years, women’s participation in basketball, a very popular sports played worldwide, has rapidly increased (Garbenytė-Apolinskienė et al., 2019). Although physical and physiological requirements for male basketball players are higher than in female, they seem to experience similar activity demands (Stojanović et al., 2018). To date little is known about sex-related differences in sports adaptations. Hunter et al. (Hunter, 2014, 2016) described differences between male and female basketball players in recovery attributing them principally to the speed of contraction, muscle strength, muscle groups involved, muscle perfusion, skeletal muscle metabolism, and fiber type properties. Physical training biology is complex to study because it involves numerous interactions among cells, tissues, organs, systems, with remarkable cross-talk among them (Ruegsegger and Booth, 2018). Since protein composition represents the functional status of the biological process at a given time in cells, tissues, and organs (Gamberi et al., 2016) we below that proteomics of exercise allows the identification of biomolecules network that changes with training. This makes it possible to also identify and monitor (new) biomolecules that may provide a general analysis of an athlete’s condition to prevent overtraining/overreaching syndrome and improve sports performance.

The aim of the present study is to identify sex-related sports adaptation proteins in female and male basketball players using proteomics approach on plasma samples withdrawn from athletes during in-season training period but far from a competition. The results obtained will provide more information about biological processes involved during training and will help to understand adaptation to endurance training in male and female skeletal muscle. Moreover, we can identify proteins useful to understand training levels in male and female athletes.

## Materials and Methods

Unless specified, all reagents were obtained from Sigma (St. Louis, MO, United States).

### Participants

A cohort of 20 professional basketball players, 10 female (BF) and 10 male (BM), and 20 sedentary: 10 male (CM) and 10 female (CF) as control, were involved in this study. The control groups were recruited among sedentary students of the degree course in Motor Sciences, Sport and Health of the University of Florence. The athletes were recruited among the male and female local sports clubs in Florence “US AFFRICO-Firenze,” and they have been practicing this sports for more than 5 years.

They trained at least five times a week according to specific training programs with sessions lasting 2 h per day. The training protocol involved for both male and female teams concerning technical and aerobic exercise is previously reported (Militello et al., 2021) and resumed as follows: half an hour of the low-moderate run followed by interval training runs with sprinting and repeating. Then work on shoulder muscles by performing the classic front and side lifts with weightlifting dumbbell and work with abdominals essential to maintain balance in every movement.

Weight is measured to the nearest 0.1 kg and height to the nearest 0.5 cm, body mass index (BMI) was calculated from the ratio of body weight (kg) to body height (m^2^). A medical history and physical activity questionnaire were completed by participants to determine the eligibility: all subjects were adults (≥20 years) and of Caucasian ethnicity, none of them used antioxidant or nutritional supplements and were selected by non-smoking status, age, and stable body weight. All women were enrolled randomly respected to the menstrual cycle. All participants received a complete explanation of the purpose, risks, and procedures of the study. Written informed consent was provided prior to enrolment in the study that was conducted according to the policy statement outlined in the Declaration of Helsinki and approved by the Local Ethics Committee of the Florence University, Italy (AM_Gsport 15840/CAM_BIO).

### Handling of Plasma Samples and Albumin and IgG Depletion

A capillary blood sample (300 μl) was taken using a heparinized Microvette CB300 (Sarstedt, Nümbrecht, Germany) from the finger of each volunteer as previously reported (Militello et al., 2021). To obtain plasma the blood tubes were immediately centrifuged for 10 min at 2,000 × *g* using a table centrifuge. Plasma samples were stored at −80°C in the freezer and the experiments were done on stored samples.

All the biological samples were collected in the morning and as regards the athletes, away from sports competition and before the daily training sessions. Four different pools of plasma samples, one for each group of participants (10 participants for each group) were prepared as follows: basket male (BM), basket female (BF), control male (CM), and control female (CF). It is known that in plasma Albumin and IgG are abundant proteins interfering with proteomic studies because they are present over a wide pI and molecular weight range. For this, it was carried out a depletion of albumin and IgG on each pooled sample using the Aurum™ Serum Protein Mini Kit (Bio-Rad Laboratories, Hercules, CA, United States). Briefly, the Aurum serum protein mini-column (200 μl Affi-Gel^®^ Blue and Affi-Gel protein A, Bio-Rad Laboratories, Hercules, CA, United States) was washed with 25 mM phosphate buffer (pH 7). The plasma sample (60 μl) was mixed with 200 μl of serum protein binding buffer, was loaded on the column, and incubated for 10 min, with intermittent vortexing. The column was centrifuged for 1 min at 6,000 rpm and the eluate was collected. The column was then washed with 200 μl of serum protein binding buffer, vortexed, and centrifuged. The eluate was collected and combined with the first eluate to form the unbound fraction.

The unbound fraction obtained was precipitated with ice-cold acetone (1:4) overnight at −20°C. Samples were then centrifuged at 12,000 rpm for 30 min and acetone was decanted. The pellets were suspended with 120 μl of rehydration solution 8 M urea, 4% (w/v) CHAPS, 50 mM DTT. Total protein contents were obtained using the Bradford assay.

### Two-Dimensional Polyacrylamide Gel Electrophoresis

The 2-DE experiments were performed in triplicate for each pool of plasma. Isoelectric focusing (IEF) was carried out on 11 cm IPG-strips pH 3–10 NL (Bio-Rad Laboratories, Hercules, CA, United States) and achieved using Protean^®^ i12TM IEF System (Bio-Rad Laboratories, Hercules, CA, United States). A total of 20 μg of protein sample for analytical and 200 μg for preparative gels, were loaded on the strips and actively rehydrated (at 50 V), for 16 h, in 200 μl rehydration solution (8 M urea, 4% (w/v) CHAPS, 50 mM DTE) supplemented with 0.5% (v/v) carrier ampholyte (Bio-Rad Laboratories, Hercules, CA, United States) and a trace of bromophenol blue. The strips were then focused at 16°C according to the following electrical conditions: 250 V for 20 m (rapid), from 250 V to 8,000 V for 1 h, 8,000 V until a total of 23,000 V/h was reached, with a limiting current of 50 μA/strip. After focusing, analytical and preparative IPG strips were equilibrated for 10 min in 6 M urea, 30% (v/v) glycerol, 2% (w/v) SDS, 2% (w/v) DTT in 0.05 M Tris-HCl buffer, pH 6.8, and subsequently for 10 min in the same buffer solution where DTT was substituted with 2.5% (w/v) iodoacetamide. IPG strips were then placed on top of 9–16% polyacrylamide linear gradient gels (18 cm × 20 cm × 1.5 mm) and embedded in 0.5% heated low-melting agarose in SDS electrophoresis running buffer (25 mM Tris, 192 mM glycine,0.1% (w/v) SDS, pH 8.3). SDS-PAGE was performed in a PROTEAN II xi cell gel electrophoresis unit (Bio-Rad Laboratories, Hercules, CA, United States) at 200 V until the dye front reached the bottom of the gel. Analytical gels were stained with ammoniacal silver nitrate as previously described (Guidi et al., 2011) and preparative gels were stained with colloidal Coomassie blu G-250 (Pietrovito et al., 2015).

### Image Analysis and Statistics

Two-DE images were scanned by using the Amersham Imager 600 (GE Healthcare, Chicago, IL, United States). For each investigated group, namely, BM, BF, CM, and CF, three technical replicates were performed. The gel images were saved with a resolution of 300 dpi and in 16-bit TIFF format.

Image analysis was carried out using the Progenesis SameSpots software version 4 (Nonlinear Dynamics, Newcastle, United Kingdom), which allows spot detection, background subtraction, and protein spot volume quantification. The gel image showing the highest number of spots and the best protein pattern was chosen as the reference image and its spots were then matched across all gels. This reference image was used to quantify and normalize the spot volumes. Statistical analysis was performed using default parameters of the Progenesis SameSpots Stat module.

The univariate data analysis was performed as one-way ANOVA and the differentially expressed spots (ANOVA *p*-value < 0.05) were subsequently analyzed by Tukey’s multiple comparisons tests using GraphPad Prism software version 6 (GraphPad Software, San Diego, CA, United States) to find out the significant differences between groups.

### Protein Identification by Liquid Chromatography-Mass Spectrometry

In this experiment, 20 spots were manually excised from Coomassie-stained preparative gels and were subjected to a protocol of *in situ* digestion. Briefly, each gel piece was de-stained with three cycles of 0.1 M NH_4_HCO_3_ of pH 8 and acetonitrile (ACN), followed by reduction (10 mM DTT in 100 mM NH_4_HCO_3_) at 56°C for 45 min, and alkylation (55 mM IAM in 100 mM NH_4_HCO3) at RT for 30 min. Finally, the gel plugs were rehydrated in 40 μl sequencing grade modified trypsin (10 ng ml^−1^ trypsin; 10 mM NH_4_HCO_3_) and incubated overnight at 37°C. Peptide mixtures were eluted and washed by using 0.1% formic acid and ACN, vacuum-dried, and resuspended in 0.1% formic acid for the subsequent liquid chromatography-tandem mass spectrometry (LC-MS/MS) analysis. Peptide mixtures were analyzed by a 6520 Accurate-Mass Q-TOF LC/MS system (Agilent Technologies, Santa Clara, CA, United States) equipped with a 1200 HPLC system and a chip cube (Agilent Technologies, Santa Clara, CA, United States). After loading, the peptide mixture (1 μl) was concentrated and desalted at a flow rate of 4 μl/min in a 40 nl enrichment column with 0.1% HCOOH as eluent. The sample was then fractionated on a C18 reverse-phase capillary column (75 μm × 43 mm in the Agilent Technologies chip, Santa Clara, CA, United States) at a flow rate of 400 nl/min, with a linear gradient of eluent B (0.1% HCOOH in 95% ACN) in A (0.1% HCOOH in 2% ACN) from 5 to 80% in 50 min.

Peptide analysis was performed using the data-dependent acquisition of one MS scan (mass range m/z 300--2,400) followed by an MS/MS scan of the five most abundant ions in each MS scan. MS/MS spectra were measured automatically when the MS signal was greater than the threshold of 50,000 counts. Double, triple, and quadruple charge ions were preferably isolated and fragmented over singly charged ions. Data were acquired through Mass Hunter software (Agilent Technologies, Santa Clara, CA, United States). The acquired data, containing MS and MS/MS spectra, were transformed in. mgf format and used for protein identification with a licensed version of Mascot Software (London, United Kingdom^1^). Mascot search parameters included: NCBI as database; Homo sapiens as taxonomy; trypsin as an enzyme, allowed number of missed cleavage 3; carbamidomethyl, C as fixed modifications; oxidation of methionine (Met), pyro-Glu (N-terminal Gln and Glu) as variable modifications, peptide charge from +2 to +4. Mass tolerance was set to 10 ppm while MS/MS tolerance to 0.2 Da. Every protein was selected as significant when at least 1 peptide displayed a *p*-value < 0.05.

### Western Blot Analysis

Plasma protein samples (10 μg) were added to 4× Laemmli buffer (0.5 M Tris-HCl pH 6.8, 10% SDS, 20% glycerol, β-mercaptoethanol, 0.1% bromophenol blue) and boiled for 5 min. The samples were separated by 15% SDS-PAGE and transferred onto polyvinylidene fluoride membrane (PVDF) using the Trans-Blot Turbo Transfer System (Bio-Rad Hercules, CA, United States). Western blot was performed using a monoclonal antibody against Dermcidin (sc-33656) and Transthyretin (sc-377517) all provided by Santa Cruz Biotechnology (Santa Cruz Biotechnology, Santa Cruz, CA, United States). After incubation with horseradish peroxidase (HRP)-conjugated anti-mouse IgG (1:10,000) (Santa Cruz Biotechnology, Santa Cruz, CA, United States), immune complexes were detected with the enhanced chemiluminescence (ECL) detection system (GE Healthcare, Chicago, IL, United States) and by the Amersham Imager 600 (GE Healthcare, Chicago, IL, United States).

The amount of each band was quantified with densitometric analysis performed using the ImageJ image processing program^2^ and normalized using the total protein amount of the corresponding lane detected on PVDF membranes stained with Coomassie brilliant blue R-250 as previously reported (Militello et al., 2021). Statistical analysis of the data was performed by Student’s *t*-test using GraphPad Prism software (GraphPad Software, San Diego, CA, United States); *p*-value < 0.05 was considered statistically significant.

## Results

Descriptive characteristics of the participants were reported in Table 1. In summary, mean age of the subjects involved in this study was 24.4 ± 4.2 years. Male and female basketball players were taller than the respective controls, but no significant differences were found between the players and controls groups regarding BMI values (*p*-value = 0.59 for male, *p*-value = 0.94 for female). Significant differences were found in characteristics between male and female; male subjects were weightier and taller than the respective female subjects both in basketball players and controls groups but also in this case there were no significant differences in BMI between men and women (*p*-value was 0.2 for basketball players and 0.45 for control groups).

**TABLE 1:** Participants’ characteristics.

Characteristics	Mean (SD)				Unpaired *t*-test^a^			
	Basket male	Control male	Basket female	Control female	BM *vs.* CM	BF *vs.* CF	BM *vs.* BF	CM *vs.* CF
Age (year)	21 ± 2.2	26.1 ± 4.1	25.1 ± 5.5	26.9 ± 2.2	0.001**	0.42	0.03*	0.64
Weight (kg)	81.5 ± 10.2	73 ± 8.7	68.7 ± 11.9	58.7 ± 5.8	0.05	0.051	0.02*	0.001**
Height (cm)	186 ± 0.06	178.7 ± 0.06	175.6 ± 0.08	163.4 ± 0.06	0.008**	0.004**	0.004**	< 0.0001****
BMI (kg/m^2^)	23.6 ± 2.7	22.9 ± 2.9	22.1 ± 2.05	22 ± 2.3	0.59	0.94	0.2	0.45

^a^Unpaired t-test was performed by GraphPad Prism software version 6 between male basket group (BM), male control group (CM), female basket group (BF), and female control group (CF): (*p < 0.05), (**p < 0.01), (***p < 0.001), and (****p < 0.0001).

### Overview of Plasma Protein Profiles

**FIGURE 1:**
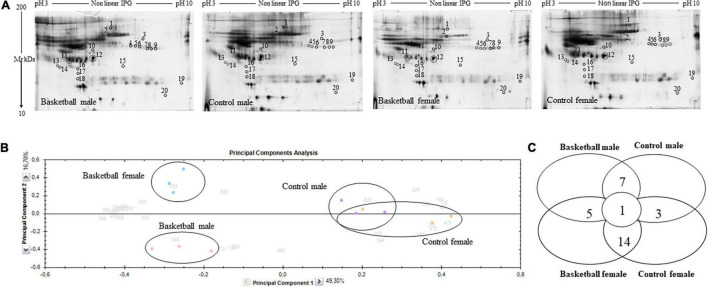
Proteomic profile of Basketball players and controls **(A)** Representative 2-DE images of silver-stained gels of plasma proteins run on NL pH 3–10 IP strip and in 9–16% polyacrylamide linear gradient. Circles and numbers indicate statistically differentially abundant proteins between the four groups analyzed as reported in Table 2. **(B)** Multivariate analysis of the 2-DE gel images results using principal components analysis (PCA) performed by Progenesis SameSpots software version 4. **(C)** Distribution of differentially abundant protein spots between pairwise comparisons of basketball male, control male, basketball female, and control female groups as detected by 2-DE analysis.

Protein profiles of plasma samples obtained from BM, BF, CM, and CF were analyzed by 2-DE as reported in methods. Before this analysis, we depleted plasma samples from Albumin and IgG to remove highly abundant proteins (Guidi et al., 2011). As shown in Figure 1A, the presence of traces of albumin in the sample could be due to their abundance which exceeds the depletion efficiency of the columns used. However, this was good to avoid the removal of low abundant important regulatory proteins bound to albumin. It was well known that several cytokines were significantly reduced when albumin is totally removed from plasma samples and this may confound proteomic analysis (Granger et al., 2005). To obtain statistically significant results, each plasma protein sample was run in triplicate.

After automatic spot detection, an average of about 835 protein spots were detected in each gel (Figure 1A). The computational 2-DE gel image analysis pointed out 33 differentially expressed protein spots (ANOVA *p*-value < 0.05). In addition to univariate analysis, a correlation analysis was performed to evaluate the relationships between the expression profiles of spots (Table 2).

**TABLE 2:** Quantitative data and statistical analyses of protein spots whose intensity levels significantly differed among the plasma of the four groups.

Spots	Normalized volume (arbitrary unit)^a^ Mean (SD)				ANOVA *p*-value^b^	Tukey’s test^c^/fold change^d^			
	Basket male	Control male	Basket female	Control female		BM *vs.* CM	BF *vs.* CF	BM *vs.* BF	CM vs. CF
1	2.2 ± 1	3.9 ± 0.9	2.9 ± 0.9	5.2 ± 0.5	0.032	ns	*/−1.8	ns	ns
2	22.4 ± 1.3	26.7 ± 1.9	20.5 ± 3.3	26.3 ± 1.2	0.026	ns	*/−1.3	ns	ns
3	2 ± 0.8	0.7 ± 0.1	1 ± 0.4	0.6 ± 0.03	0.009	*/+2.9	ns	ns	ns
4	20.6 ± 5.8	17.6 ± 4.9	22.2 ± 4.7	10 ± 3.2	0.025	ns	*/+2.2	ns	ns
5	31.2 ± 9	19.1 ± 5	34.7 ± 11.9	12.7 ± 3	0.010	ns	*/+2.7	ns	ns
6	17.6 ± 4.3	9.1 ± 0.4	15.8 ± 3.4	8.2 ± 2	0.005	*/+1.9	ns	ns	ns
7	24.4 ± 3.1	11.3 ± 0.6	27.6 ± 7	12.2 ± 5.5	0.013	*/ + 2.2	*/ + 2.3	ns	ns
8	25 ± 1.8	11 ± 1.8	32.3 ± 10	11.9 ± 5.3	0.007	ns	*/+2.7	ns	ns
9	21.4 ± 4	9.5 ± 2.5	27.4 ± 9.1	9.6 ± 5	0.019	ns	*/+2.8	ns	ns
10	0.9 ± 0.4	0.5 ± 0.05	0.4 ± 0.3	2 ± 0.5	0.006	ns	**/−5	ns	**/−4
11	76.4 ± 10	88.3 ± 7	61.9 ± 7	89.8 ± 17.2	0.035	ns	*/−1.4	ns	ns
12	83.6 ± 6.5	118.3 ± 10.7	72.4 ± 7.8	110.3 ± 8	0.0004	**/−1.4	**/−1.5	ns	ns
13	3.9 ± 0.3	0.9 ± 0.3	1 ± 0.3	2 ± 0.5	0.001	****/+4.3	*/−2	****/+3.9	*/−2.2
14	6.3 ± 0.5	1.9 ± 0.9	1.5 ± 0.3	4.2 ± 1.5	0.001	**/+3.3	*/−2.8	**/+4.2	ns
15	0.4 ± 0.1	0.4 ± 0.04	0.2 ± 0.05	0.5 ± 0.2	0.047	ns	*/−2.5	ns	ns
16	3.9 ± 1	2.4 ± 0.4	2 ± 0.3	2 ± 0.5	0.024	ns	ns	*/+1.9	ns
17	1 ± 0.4	0.6 ± 0.2	0.9 ± 0.09	0.3 ± 0.2	0.017	ns	*/+3	ns	ns
18	10.2 ± 4	5.5 ± 0.7	4.8 ± 0.3	3.9 ± 1.2	0.020	ns	ns	*/+2.1	ns
19	1.4 ± 0.9	2.9 ± 0.4	2.8 ± 1.1	0.8 ± 0.5	0.027	ns	ns	ns	*/+3.6
20	0.1 ± 0.05	0.04 ± 0.007	0.05 ± 0.03	0.7 ± 0.1	0.017	*/+2.5	ns	*/+2	ns

*^a^The mean of normalized spot volume and the respective SD were calculated by the GraphPad Prism software version 6 using the normalized volume data calculated by the Progenesis SameSpots software version 4. All data were reported in order of magnitude 10^–6^.*

*^b^ANOVA test was performed by the Progenesis SameSpots software version 4 to determine if the relative change was statistically significant (p < 0.05).*

^c^Tukey’s post hoc test was performed on ANOVA p-values by the GraphPad Prism software version 6 (*p < 0.05), (**p < 0.01), (***p < 0.001), (****p < 0.0001), (ns = not significant).

*^d^Fold change was calculated by the GraphPad Prism software version 6. It is the ratio of the mean normalized spot volumes of the male basket group (BM), male control group (CM), female basket group (BF), and female control group (CF). It was reported only for statistically significant values.*

To obtain an overview of the proteomic data for overall trends in all groups, a multivariate analysis, principal component analysis (PCA) was performed. In the PCA biplot obtained, each point describes the collective expression profiles of one sample; gels were grouped according to the variance of protein spot abundance, so the plot demonstrated consistent reproducibility among replicate samples within each group. The PCA biplot, showed in Figure 1B, revealed four distinct main protein profile groups corresponding to (i) basketball male group (pink circle), (ii) basketball female group (blue circle), (iii) CM group (violet circle), and (iv) CF group (orange circle). The first principal component, which distinguished 49.3% of the variance, clearly separated the proteome data of basketball groups (both male and female) from controls, and the second component, with an additional 16.7% of the variance, clearly distinguished the basketball male group from basketball female group while the controls groups tended to be closer. PCA plot suggested that sports training drastically affect protein patterns where differences between male and female were more evident among the players than controls. We performed the correlation analysis on protein abundance levels. All the 33 spots, differentially expressed between the 4 groups (Figure 1C), were clustered according to how closely correlated their expression was. In particular, spots with a positive correlation value showed similar abundance profiles while proteins, which a negative correlation value showed opposing abundance profiles.

**FIGURE 2:**
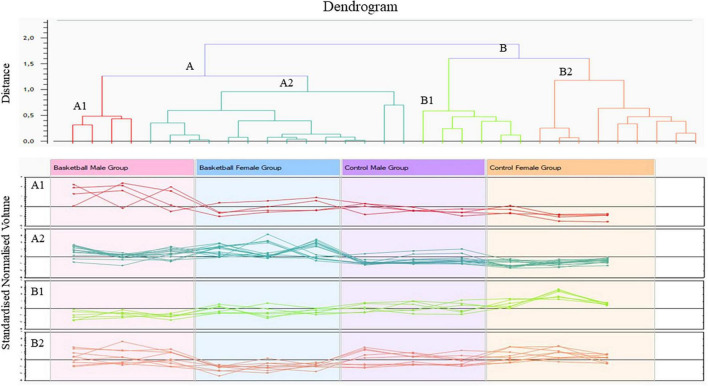
Cluster analysis of protein differentially expressed between the four groups (ANOVA *p*-value < 0.05) performed by Progenesis SameSpots software version 4.

The Progenesis SameSpots software drew a dendrogram reported in Figure 2; it showed clusters with the arrangement of spots in two different major groups: A and B. A was composed of 18 spots (54.5% of the total proteins) whose expression profile was affected by the sports activity; B was formed by 15 spots (45.5%) and showed the spots whose expression profile was differently expressed between male and female. In particular, analyzing the lower branches of the dendrogram we could identify four subgroups as shown in Figure 2; in detail, regarding group A1 the intensity of the 4 spots (12.1%) was influenced not only by sports but also by sex while in subgroups A2 the expression profile of the 14 spots (42.4%) changed exclusively by sports activity. In subgroups B1 and B2, formed respectively by 6 spots (18.2%) and 9 spots (27.3%), the expression profile varied only by sex and more precisely in subgroup B1 were grouped spots whose expression was increased in female than male and in B2 the trend was opposite showing higher expression level in male than female.

### Identification of Differentially Expressed Proteins

The comparative analysis revealed 33 protein spots differentially expressed in plasma of the four groups studied, with a *p*-value < 0.05. Tukey’s test showed that 20 of them were differentially expressed in groups that could be compared to each other: the terms of comparison were the practice of sports and sex. These spots were identified by mass spectrometry as reported in Table 2 where their relative amounts were expressed as the mean ± SD of the normalized volume and statistical analysis (*p*-value and fold change).

Among them, we found 14 proteins (70% of the total) differentially expressed in basketball female players in comparison with their relative controls, while 7 (35%) were the proteins differentially expressed in basketball male players in comparison with their controls.

Only 4 spots (20%) resulted to be differentially expressed in both male and female basketball groups compared to their controls: p532 (Q15751) (fold change > 2) increased in male and female athletes in comparison to control; on the contrary Transthyretin Chain A (P02766) decreased in athletes comparing to controls (fold change −1.4 in male and −1.5 in female). Spectrin alpha chain, non-erythrocytic 1 (Q13813), and Peroxisomal acyl-coenzyme A oxidase 1 (Q15067) were upregulated in male athletes (fold change 4.3 and 3.3 respectively) in comparison with controls and downregulated in female basketball players (fold change −2 and −2.8, respectively) in comparison with controls. Spectrin alpha chain, non-erythrocytic 1 (Q13813) was differentially expressed in all groups. In particular, it was upregulated (fold change 3.9) in male basket players in comparison to female basket players. In control groups, it was downregulated (fold change −2.2) in men compared to women.

### Proteins Differentially Expressed Among the Different Groups

Table 3 were reported the 14 proteins differentially expressed in female basketball players in comparison to the control subjects: among them, 4 were up-regulated and 10 down-regulated. In the up-regulated proteins (fold change > 2) we found Fibrinogen beta chain (P02675), Centrosome-associated protein CEP250 (Q9BV73), p532 (Q15751), and Histone H3.1 (P68431). The down-regulated spots (fold change > –1.3) identified were Dermcidin (P81605), Serotransferrin (P02787) (spot 1 and 2), Haptoglobin (P00738), Complement component C3 (P01024), Zinc-alpha-2-glycoprotein (P25311), Transthyretin (P02766), Spectrin alpha chain, non-erythrocytic 1 (Q13813), Peroxisomal acyl-coenzyme A oxidase 1 (Q15067) and RNA-binding protein 15 (Q8TF72).

**TABLE 3:** Differentially expressed protein spots from 2D-GE identified by LC-MS/MS Analysis.

Spots no^a^	Protein name	Gi number	AC^b^	Gene name	Score^c^	Protein sequence coverage %^d^	Tukey’s test^e^/fold change^f^			
							BM *vs.* CM	BF *vs.* CF	BM *vs.* BF	CM *vs.* CF
1	Serotransferrin	gi|553788	P02787	TF	123	18	ns	*/−1.8	ns	ns
1	Dermcidin	gi|16751921	P81605	DCD	64	25.5	ns	*/−1.8	ns	ns
2	Serotransferrin	gi|110590597	P02787	TF	1960	74.4	ns	*/−1.3	ns	ns
3	The human T-cell receptor	gi|553734	A0N4V7	Tcr−alpha	35	38.1	*/+2.9	ns	ns	ns
4	Fibrinogen beta chain	gi|237823915	P02675	FGB	904	82	ns	*/+2.2	ns	ns
5	Centrosome-associated protein CEP250	gi|2832237	Q9BV73	CEP250	54	2.1	ns	*/+2.7	ns	ns
7	p532	gi|1477565	Q15751	HERC1	62	1.3	*/+2.2	*/+2.3	ns	ns
11	Haptoglobin	gi|306882	P00738	HP	98	21.7	ns	*/−1.4	ns	ns
11bis	Complement component C3	gi|179665	P01024	C3	151	3.4	ns	*/−1.4	ns	ns
11ter	Zinc-alpha-2-glycoprotein	gi|38026	P25311	AZGP1	231	34.1	ns	*/−1.4	ns	ns
12	Transthyretin Chain A	gi|126030594	P02766	TTR	551	89.8	**/−1.4	**/−1.5	ns	ns
13	Spectrin alpha chain, non-erythrocytic 1	gi|179106	Q13813	SPTAN1	50	2.8	****/+4.3	*/−2	****/+3.9	*/−2.2
14	Peroxisomal acyl-coenzyme A oxidase 1	gi|458119	Q15067	ACOX1	38	5	**/+3.3	*/−2.8	**/+4.2	ns
15	RNA-binding protein 15	gi|14041646	Q96T37	RBM15	50	3.7	ns	*/−2.5	ns	ns
16	T cell receptor	gi|553734	A0N4V7	Tcr-alpha	34	38.1	ns	ns	*/+1.9	ns
17	Histone H3.1	gi|315113506	P68431	H3C1	39	59.1	ns	*/+3	ns	ns
18	SH2 domain-containing adapter protein B	gi|406738	Q15464	SHB	38	3	ns	ns	*/+2.1	ns
19	Programmed cell death protein 4	gi|224036249	Q53EL6	PDCD4	36	9.1	ns	ns	ns	*/+3.6
20	Tumor susceptibility gene 101 protein	gi|48425520	Q99816	TSG101	41	15.9	*/+2.5	ns	*/+2	ns

*^a^Spot numbers reported in the representative 2-DE images shown in Figure 1.*

*^b^Accession number in Swiss-Prot/UniProtKB (http://www.uniprot.org/).*

*^c^MASCOT MS score (Matrix Science, London, United Kingdom; http://www.matrixscience.com). MS matching score greater thank 56 was required for a significant MS hit (p-value < 0.05).*

*^d^Sequence coverage = (number of the identified residues/total number of amino acid residues in the protein sequencer) × 100%.*

^e^Tukey’s post hoc test was performed on ANOVA p-values by GraphPad Prism software version 6 (*p < 0.05), (**p < 0.01), (***p < 0.001), (****p < 0.0001), (ns = not significant).

*^f^Fold change was calculated by GraphPad Prism software version 6. It is the ratio of the mean normalized spot volumes of the male basket group (BM), male control group (CM), female basket group (BF), and female control group (CF). It was reported only for statistically significant values.*

Between the six proteins differentially expressed in men (reported in Table 3), five were upregulated (fold change > 2) in athletes in comparison to control. These were T-cell receptor (A0N4V7), p532 (Q15751), Spectrin alpha chain, non-erythrocytic 1 (Q13813), Peroxisomal acyl-coenzyme A oxidase 1 (Q15067) and Tumor susceptibility gene 101 protein (Q99816). Only one protein, Transthyretin Chain A (P02766), was down-regulated (fold change −1.4) in basketball groups in comparison to control. Among proteins differentially expressed between male and female athletes we found for all of them an increase in male in comparison with female (fold change > 1.9). The five identified proteins were: Spectrin alpha chain, non-erythrocytic 1 (Q13813), Peroxisomal acyl-coenzyme A oxidase 1 (Q15067), T cell receptor (A0N4V7), SH2 domain-containing adapter protein B (Q15464), and Tumor susceptibility gene 101 protein (Q99816). Along with the proteins differentially expressed between controls male and female, we found Spectrin alpha chain, non-erythrocytic 1 (Q13813) (fold change −2.2) which expression decrease in male in comparison with female. The protein that we found upregulated in male in comparison to female (fold change 3.6) was Programmed cell death protein 4 (Q53EL6).

### Overview of Functional Classification of Plasma Proteins Identified

**FIGURE 3:**
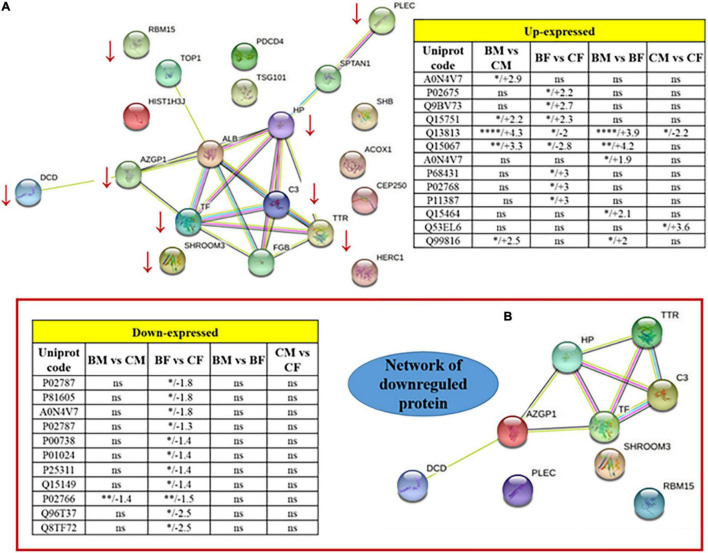
Protein-protein interaction network of the totality of proteins identified by 2D spots **(A)**. Two tables included as inserts the fold change values for up or downregulated proteins. Panel **(B)** is a zoomed visualization of the downregulated protein network. *p < 0.05, **p < 0.01, ***p < 0.001, and ****p < 0.0001.

The REACTOME and STRING pathway analyses were performed to visualize functional enrichment for the upregulated and downregulated proteins and the results of potential interactions identified were reported in Figure 3. The significantly enriched Reactome pathways were reported in Table 4. For the upregulated proteins, we found enriched pathways involving the peroxisomal-protein import, ion/eme transport, and platelet degranulation and secretion. In downregulated proteins we found pathways involved in the innate immune system, the pro-inflammatory response, and the neutrophil degranulation, all pathways critical for inflammatory damage.

**TABLE 4:** List of up/down regulated significant pathways derived from Reactome analysis (*p*-value < 0.05).

Pathway name	Entities found	Entities Total	Entities ratio	Entities *p*-Value	Entities FDR
**Upregulated proteins**					
TYSND1 cleaves peroxisomal proteins	1	7	0.001	8.42E-3	9.35E-2
Platelet degranulation	2	128	0.011	1.02E-2	9.35E-2
Response to elevated platelet cytosolic Ca2 +	2	133	0.011	1.09E-2	9.35E-2
Beta-oxidation of very long chain fatty acids	1	11	0.001	1.32E-2	9.35E-2
HDL remodeling	1	11	0.001	1.32E-2	9.35E-2
Transport of organic anions	1	12	0.001	1.44E-2	9.35E-2
Caspase-mediated cleavage of cytoskeletal proteins	1	12	0.001	1.44E-2	9.35E-2
alpha-linolenic acid (ALA) metabolism	1	13	0.001	1.56E-2	9.35E-2
alpha-linolenic (omega3) and linoleic (omega6) acid metabolism	1	13	0.001	1.56E-2	9.35E-2
p130Cas linkage to MAPK signaling for integrins	1	15	0.001	1.8E-2	9.35E-2
GRB2:SOS provides linkage to MAPK signaling for Integrins	1	15	0.001	1.8E-2	9.35E-2
Membrane binding and targeting of GAG proteins	1	15	0.001	1.8E-2	9.35E-2
Synthesis And Processing Of GAG, GAGPOL Polyproteins	1	15	0.001	1.8E-2	9.35E-2
Heme biosynthesis	1	15	0.001	1.8E-2	9.35E-2
HCMV Late Events	2	173	0.015	1.8E-2	9.35E-2
Recycling of bile acids and salts	1	16	0.001	1.92E-2	9.35E-2
Heme degradation	1	16	0.001	1.92E-2	9.35E-2
MyD88 deficiency (TLR2/4)	1	19	0.002	2.27E-2	9.35E-2
IRAK4 deficiency (TLR2/4)	1	20	0.002	2.39E-2	9.35E-2
Regulation of TLR by endogenous ligand	1	21	0.002	2.51E-2	9.35E-2
**Downregulated proteins**					
Post-translational protein phosphorylation	2	107	0.009	3.65E-3	6.7E-2
Alternative complement activation	1	5	0	4.31E-3	6.7E-2
Regulation of Insulin-like Growth Factor (IGF) transport and uptake by Insulin-like Growth Factor Binding Proteins (IGFBPs)	2	124	0.011	4.87E-3	6.7E-2
Activation of C3 and C5	1	7	0.001	6.02E-3	6.7E-2
Neutrophil degranulation	3	480	0.041	6.85E-3	6.7E-2
Type I hemidesmosome assembly	1	11	0.001	9.45E-3	6.7E-2
Caspase-mediated cleavage of cytoskeletal proteins	1	12	0.001	1.03E-2	6.7E-2
Retinoid cycle disease events	1	13	0.001	1.12E-2	6.7E-2
Diseases associated with visual transduction	1	13	0.001	1.12E-2	6.7E-2
Diseases of the neuronal system	1	13	0.001	1.12E-2	6.7E-2
Innate Immune System	4	1,19	0.103	1.4E-2	7.02E-2
The canonical retinoid cycle in rods (twilight vision)	1	23	0.002	1.97E-2	7.93E-2
Miscellaneous transport and binding events	1	26	0.002	2.22E-2	7.93E-2
Purinergic signaling in leishmaniasis infection	1	27	0.002	2.31E-2	7.93E-2
Cell recruitment (pro-inflammatory response)	1	27	0.002	2.31E-2	7.93E-2
Extracellular matrix organization	2	301	0.026	2.64E-2	7.93E-2
Transferrin endocytosis and recycling	1	31	0.003	2.64E-2	7.93E-2
Apoptotic cleavage of cellular proteins	1	38	0.003	3.23E-2	8.8E-2
Retinoid metabolism and transport	1	44	0.004	3.73E-2	8.8E-2
Metabolism of fat-soluble vitamins	1	48	0.004	4.07E-2	8.8E-2

**FIGURE 4:**
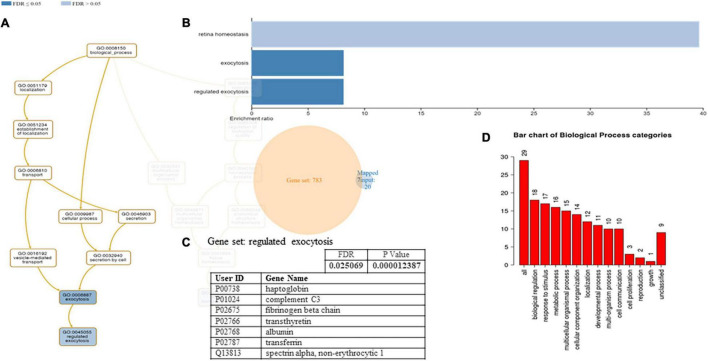
Identified proteins and Gene Ontology (GO) analysis (Biological process categories). **(A)** DAG of all the proteins identified. **(B)** Bar Chart with enrichment ratio and FDR. **(C)** Enriched Gene set showing the distribution of the identified proteins. **(D)** Bar chart showing the Biological Process to which the identified proteins belong.

**FIGURE 5:**
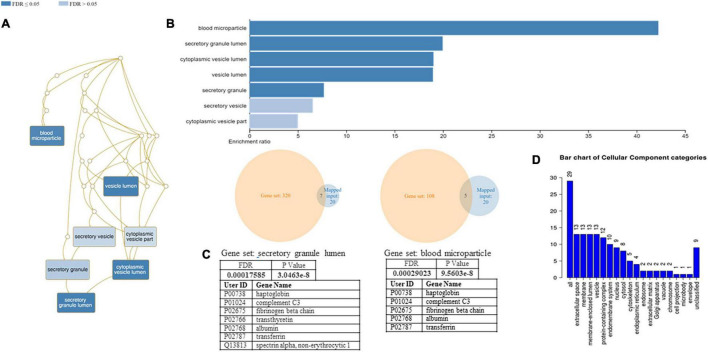
Identified proteins and GO analysis (Cellular Component categories). **(A)** DAG of all the proteins identified. **(B)** Bar Chart with enrichment ratio and FDR. **(C)** Enriched Gene set showing the distribution of the identified proteins. **(D)** Bar chart showing the Cellular Component to which the identified proteins belong.

A functional classification analysis using the webgestal^3^ was performed using the whole list of identified proteins. The classification terms Biological process and Cellular Component according to the Gene Ontology (GO), show that proteins identified in plasma mostly belong to the extracellular space component confirming their secretory origin, in accordance with the provenience of the samples. In particular Biological process categories reported in Figure 4 showed directed acyclic graph (DAG panel a) involving exocytosis and regulated exocytosis with significant false discovery rate and *p*-value (panel b). In Figure 4 panel c was shown the enriched gene set: regulated exocytosis. Figure 5 was reported the GO classification Cellular component and the enrichment analysis showed a directed acyclic graph (DAG panel a) involving secretory granule and cytoplasmic vesicle lumen with corresponding significant false discovery rate and *p*-value (panel b). As expected, the GO enrichment analysis also highlighted the gene set enrichment: blood microparticles. In Figure 5 panel c was shown the identified proteins that belong to the corresponding gene sets: secretory granule lumen and blood microparticle.

### Validation of Proteomic Results

We performed a western blot analysis in plasma samples of all groups to validate the proteomics data and to confirm our biological hypothesis. In particular, we validated Dermcidin to deepen clarify muscle crosstalk under training and Transthyretin, a possible biomarker of hypermetabolic state in athletes.

**FIGURE 6:**
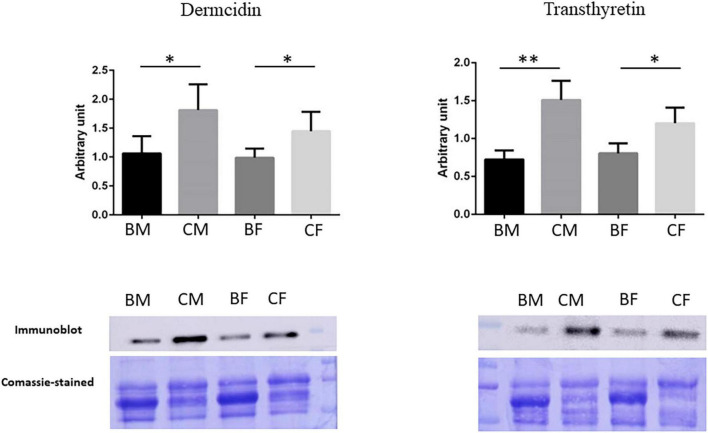
Validation of proteomic results. Histograms and representative immunoblot images of Dermcidin and Transthyretin in BM (basketball male group), CM (control male group), BF (basketball female group), and CF (control female group). Normalization of immunoblot was performed on Coomassie-stained PVDF membrane. The statistical analysis was carried out by the two-tailed *t*-test using Graphpad Prism 8 (*p < 0.05; **p < 0.01).

The results were reported in Figure 6: the intensities of immunostained-detected bands were normalized on the total quantity of proteins using the same blot stained with Coomassie brilliant blue. The histograms showed the normalized mean of results ± SD. The significance of changes was analyzed by Student’s *t*-test using GraphPad Prism software.

Validation for Transthyretin confirms its down-regulation in basketball players male and female as reported in proteomics data. The statistical analysis showed a reduction of 52.1% *p*-value = 0.001 (fold change −2.09) in male athletes comparing to their controls while a reduction of 33.2% *p*-value = 0.017 in female athletes in comparison control (fold change −1.5).

Proteomics results demonstrated in female basket athletes a reduction of Dermcidin value while no significant differences were reported in male groups. Western blot results confirmed these results in female athletes, with a reduction of 31.8% (*p*-value = 0.047) in comparison to the corresponding controls (fold change −1.47). A reduction in Dermcidin expression was revealed in western blot analysis from male athletes (41.3%; *p*-value = 0.031) in comparison to their control (fold change −1.7). In our proteomic experiments, we decided on a *p*-value threshold lower than 0.05 for statistical significance. Then, concerning the protein Dermcidin in male athletes and controls, it had an ANOVA *p*-value = 0.1491 and *t*-test *p*-value = 0.0967 making us conclude that there was no significant difference.

To explain the discrepancy between western validation and proteomics data we reported what was discussed by Handler et al. (2018) about the pros and cons of western blotting as a validation approach. According to these authors, in proteomics data, ANOVA and *t*-test were not all that should be considered. In our specific experiment, we have to consider that Dermcidin was present in several isoforms. Hence, quantification based only on staining intensity in 2-DE gels may be inaccurate for proteins present in several isoforms. In these specific conditions western blot technique was useful when, as in our conditions, the antibodies were of good quality.

## Discussion

In this study, we show that regular training modulates the secretion of biological material and induces metabolic changes in organisms to establish a new dynamic equilibrium. The molecular mechanisms that promote crosstalk among organs (Safdar et al., 2016) and orchestrate the positive effects of exercise is still unclear. However, exercise induces tissue adaptations through inter-tissue communication from skeletal muscle and other organs by secreting soluble factors, such as enzymes, cytokines, chemokines, hormones, and growth factors, traditionally regulated by a protein-based signaling system (Bay and Pedersen, 2020) or using small membranous extracellular vesicles (EVs) called “exerkines” (Bay and Pedersen, 2020; Thyfault and Bergouignan, 2020; Ciferri et al., 2021; Darkwah et al., 2021).

Our results confirm that regular training modulates the secretion of biological material. We found according to the GO analysis that proteins identified in plasma mainly belong to the extracellular space component confirming their secretory origin, involving both exocytosis, such as transthyretin, that can exocytose *via* exosomes (Lee et al., 2019) and regulated exocytosis, such as Tumor susceptibility gene 101 protein an exosome pathway regulator (Peng et al., 2019). Although, the enrichment analysis shows secretory granule and cytoplasmic vesicle lumen we found also other proteins like serotransferrin, an iron-binding transport protein (Gomme et al., 2005) that act beyond exosomes.

### Proteins Involved in Skeletal Muscle Adaptation

We found in plasma from baskets male and female a reduced expression of Dermcidin, a skeletal muscle myokine known to promote apoptosis under hypoxic conditions (Esposito et al., 2015). Moreover, in a recent study, Corasolla Carregari et al. (2020) found Dermcidin upregulated in muscular dystrophy, indicating this skeletal myokine as a possible candidate as a circulating biomarker of disease.

Among the proteins with a reduction in expression level in plasma of female athletes in comparison to sedentary conditions, we identify the RNA-binding protein 15 involved in skeletal muscle adaptation to exercise as reported by Van Pelt et al. (2019). With our results, we confirm that the role of transcription factors may be a critical regulatory node in adaptive responses to muscle contraction by modulating different factors (Van Pelt et al., 2019). Moreover, RNA binding proteins (RBPs) control alternative splicing and polyadenylation in skeletal muscle (Pedrotti et al., 2015).

In plasma from BF, we found an increase of Histone H3.1 which is the core component of nucleosome playing a central role in transcription regulation. These results are in line with what Lim et al. (2020) reported that training causes epigenetic changes in skeletal muscle and affects both gene expressions and histone modifications.

### Proteins Involved in Inflammation

We found a reduced expression of several secretion proteins, normally found in plasma, that play a role in modulating the expression of several acute-phase proteins (APPs): Complement C3 that acts as a chemoattractant for neutrophils in chronic inflammation; Haptoglobin that acts also as an antioxidant and Hemoglobin/haptoglobin complexes which are rapidly cleared through an endocytic degradation pathway.

In addition to these proteins, we found the adipokine Zinc-alpha-2-glycoprotein (ZAG) which is known to stimulate lipid degradation in adipocytes and recently Fan et al. (2021) suggest that it might influence lipid-related metabolism in skeletal muscle through the β-adrenergic system signaling pathway. Moreover, ZAG is downregulated by pro-inflammatory mediators and is described as having an anti-inflammatory function (Mracek et al., 2010). The reduction level of these proteins in the plasma of female athletes confirms what was reported in our previous study (Militello et al., 2021). Furthermore, we previously found in the same female athletes a reduced salivary level of cortisol. We suggest that the low level of oxidative stress in female athletes could be maintained by the high levels of plasma adiponectin present in comparison with male (Rak et al., 2017) and by the anti-inflammatory action of the protein ZAG.

Finally, we found as down regulated two rapid turnover proteins, Serotransferrin and the thyroid hormone-binding protein Transthyretin Chain A (Dellière and Cynober, 2017) many exercise-related factors, including inflammation, are able to influence their expression. Moreover, Tsunekawa et al. (2021) reported that these variables were inversely correlated with skeletal muscle mass. Athletes with higher levels of muscle mass had significantly lower levels of these proteins in comparison to controls. In this study, we found a reduced expression of protein Transthyretin also in male athletes suggesting that, as reported by Tsunekawa’s study, this protein could be considered a biomarker of hypermetabolic state in athletes with high levels of skeletal muscle mass.

### Fibrinogen and p532 (E3 Ubiquitin-Protein Ligase HERC1) Are Upregulated in Female Athletes

Among the protein showing an increase in a female basket in comparison to sedentary control, we identified the Fibrinogen beta chain and the protein p532 (E3 ubiquitin-protein ligase HERC1) which is involved in protein degradation. In a previous study (Guidi et al., 2011), we found that Fibrinogen, a protein susceptible to oxidation and the best indicator of oxidative stress, showed a reduced carbonylation level after physical activity and we concluded that this could be due to proteasome-dependent degradation. In this study, we found an increase in Fibrinogen plasma level and a contemporary increase in p532 a protein involved in the pathway protein ubiquitination (Ali et al., 2021). We can suggest that the reduced level of carbonylated Fibrinogen previously found in trained athletes was probably due to the simultaneous expression of the protein p532. The increase in plasma level of fibrinogen in athletes compensates for the degradation of the oxidized protein that is sent to the proteasome-dependent degradation system.

### Proteins Differentially Expressed in Male and Female Basketball Players

Interestingly, among the identified proteins there are five overexpressed in male respect to female players: Spectrin and Tumor susceptibility gene 101 which are involved in secretion and regulation of vesicles; SH2 domain-containing adapter protein B and the T cell receptor that take place in immune response; acyl-coenzyme A oxidase peroxisomal which is involved in lipid metabolism.

Tumor susceptibility gene 101 is a component of the ESCRT-I complex, a regulator of the vesicular trafficking process, and is required for the sorting of endocytic ubiquitinated cargos into multivesicular bodies (Stefani et al., 2011). It may also play a role in the extracellular release of microvesicles that differ from the exosomes (Nabhan et al., 2012). Moreover, Spectrin, involved in secretion, is a ubiquitous cytoskeletal protein and its proteolysis leads in several cells to the biogenesis of plasma membrane EVs (Ranganathan et al., 2021). The relationship between membrane and cytoskeleton provides structural stability and one of the proteins involved in this stability is Spectrin. A reduction of Spectrin may be an indicator of cells with a higher propensity to vesiculate (Taylor et al., 2021).

SH2 domain-containing adapter protein B and the T cell receptor are two proteins implicated in the immune response. SH2 domain-containing adapter protein B is an adapter protein that regulates several signal transductions cascades by linking activated receptors to downstream signaling components, such as FGFR1, VEGFR2, PDGFR, and T-cell antigen receptor signaling. Interestingly we found overexpressed in male players the T cell receptor also, which is an upstream component of the same pathways confirming the ability of physical exercise to significantly alters the immune system (Nieman and Wentz, 2019). Our findings agree with previous epidemiological results showing that the women have a greater Illness risk during international competition events suggesting that immune activation may be a male-specific pathway through which exercise confers benefit (Nieman and Wentz, 2019; Casaletto et al., 2020).

Finally, a protein expressed in an opposite way between male and female athletes is the acyl-coenzyme A oxidase peroxisomal, the first enzyme involved in the peroxisomal β-oxidation system. In a recent study, Bjørndal et al. (2018) suggested that fatty acid β-oxidation directly affects mitochondrial respiratory capacity in the liver. Cortright et al. (Cortright and Koves, 2000) reported that female differ from male in the mechanisms of energy homeostasis and energy metabolism. The regulation of skeletal muscle metabolism most likely involve sex steroids and new discoveries may provide further explanations for the observed sex differences in substrate utilization during training and this could influence the different mechanism of adaptation of the muscle to training in male and female.

## Conclusion

In conclusion, in our specific conditions, we identify in regular trained female athletes a reduction in expression of several proteins related to skeletal muscle adaptation and chronic inflammation confirming the anti-inflammatory effect of regular training in female muscle metabolism. Moreover, in both male and female athletes, we found a decrease in Transthyretin a cytoskeletal protein secreted through exosome and involved in muscle homeostasis and regeneration, and Dermcidin a stress-induced myokine linked to inflammatory events due to metabolic disorders, confirming the positive effects of regular training in metabolism.

The overall data obtained with Transthyretin and Dermcidin are interesting from a clinical point of view, also. Serum Transthyretin levels increase in obese people and subjects with impaired glucose tolerance/type 2 diabetes (Klöting et al., 2007; Lim et al., 2008), while has been known that athletes have an increased insulin sensitivity (Corcoran et al., 2007). The recent work by He et al. (2021) demonstrated that physical exercise reduces TTR plasma levels and its muscle receptor in obese mice, increasing insulin sensitivity. Furthermore, we found decreased levels of dermcidin, in particular in female players. Dermcidin is associated with the suppression of insulin production-release from the liver/pancreas through the inhibition of GLUT-4 (Bhattacharya et al., 2017). Therefore, the confirmed low levels of Transthyretin and Dermcidin in athletes suggest a role of these proteins in adaptative response to sports and two potential new targets for treating insulin resistance.

Further studies will confirm the presence of these proteins in EV in elite athletes and it will be interesting to fully understand the role of different isoforms of Dermcidin in male and female skeletal muscle contraction.

## Data Availability Statement

The datasets presented in this study can be found in online repositories. The names of the repository/repositories and accession number(s) can be found below: PRIDE, PXD031050.

## Ethics Statement

The studies involving human participants were reviewed and approved by Local Ethics Committee of the Florence University, Italy (AM_Gsport 15840/CAM_BIO). The patients/participants provided their written informed consent to participate in this study.

## Author Contributions

AM: conceptualization and formal analysis. RM, GP, AI, SL, and FM: methodology and investigation. AA, PM, and AM: data curation, writing, review, and editing. All authors contributed to the article and approved the submitted version.

## Conflict of Interest

The authors declare that the research was conducted in the absence of any commercial or financial relationships that could be construed as a potential conflict of interest.

## Publisher’s Note

All claims expressed in this article are solely those of the authors and do not necessarily represent those of their affiliated organizations, or those of the publisher, the editors and the reviewers. Any product that may be evaluated in this article, or claim that may be made by its manufacturer, is not guaranteed or endorsed by the publisher.

## Footnotes

1
http://www.matrixscience.com

2
imagej.nih.gov/ij/index.html

3
http://www.webgestalt.org/
